# Two Small Extracellular Vesicle sRNAs Derived From *Mycobacterium tuberculosis* Serve as Diagnostic Biomarkers for Active Pulmonary Tuberculosis

**DOI:** 10.3389/fmicb.2021.642559

**Published:** 2021-04-15

**Authors:** Geng Lu, XinRui Jiang, Anni Wu, Jiawei Zhou, Hengjun Liu, Fei He, Qiuling Zhang, Ke Zen, Shuangshuang Gu, Jun Wang

**Affiliations:** ^1^Department of Emergency, Nanjing Drum Tower Hospital, The Affiliated Hospital of Nanjing University Medical School, Nanjing, China; ^2^School of Life Sciences, Nanjing University, Nanjing, China

**Keywords:** biomarker, sRNA, serum, infection, tuberculosis

## Abstract

The rapid diagnosis of tuberculosis (TB) is of great significance for the control and treatment of TB. However, TB remains a major healthy, social, and economic burden worldwide because of the lack of ideal diagnostic biomarkers. *Mycobacterium tuberculosis (M. tuberculosis)-*encoded small RNA (sRNA) is a class of regulation small RNA. Several studies have identified *M. tuberculosis* encoded-sRNAs in the serum/plasm of *M. tuberculosis-*infected patients. Small extracellular vesicles are small membrane vesicles secreted by many cell types during physiological and pathological conditions. Recent evidence has indicated that most of the nucleic acids in the serum/plasma are packaged in the small extracellular vesicles and could serve as ideal diagnostic biomarkers. In this study, we attempted a novel approach for TB diagnosis: targeting small extracellular vesicles *M. tuberculosis* encoded sRNA (sRNA) by qRT-PCR. The results showed that *M. tuberculosis-*encoded ASdes and MTB-miR5 only existed in tuberculosis patients and have the potential to serve as a sensitive and accurate methodology for TB diagnosis.

## Introduction

Tuberculosis (TB), which is caused by *Mycobacterium tuberculosis* (*M. tuberculosis*) infection, remains a major global infectious disease with mortality among the highest (WHO, 2020). According to the data of the World Health Organization (WHO) in 2019, there were over 10 million new cases and 1.4 million patients died from this disease (WHO, 2020). The rapid diagnosis of TB is of great significance for the control and treatment of TB (WHO, 2020). To date, the imaging inspection, bacteriological inspection, molecular biological detection, and immunological experimental inspection are the most commonly used diagnosis methods for *M. tuberculosis*. However, all of these approaches have a myriad of drawbacks (2, 3), and it’s an urgent to screen novel biomarkers to improve diagnosis.

Bacterial small RNA (sRNA) is a class of post-transcriptional regulation RNA molecules (Fu et al., 2018). Several studies have identified that *M. tuberculosis* could encode sRNA (Singh et al., 2015; Lv et al., 2017; Fu et al., 2018; Chakrabarty et al., 2019; Lyu et al., 2019). These *M. tuberculosis*-encoded sRNA play an important role in post-transcriptional regulatory networks during *M. tuberculosis* infection, mainly through base pairing with mRNA and combining with protein directly (Masse et al., 2003; Gottesman, 2005; Repoila and Darfeuille, 2009; Wagner, 2009; Singh et al., 2015; Felden and Bouloc, 2017). Recently, several reports have suggested that *M. tuberculosis* encoded sRNA could be secreted into plasma/serum and have the possibility to serve as non-invasive biomarkers for diagnosis of *M. tuberculosis* infection (Fu et al., 2018; Chakrabarty et al., 2019). Chakrabarty et al. (2019) found MTBmiR-5, a miRNA encoded by *M. tuberculosis*, significantly increased in the serum of pulmonary tuberculosis and extra pulmonary tuberculosis, and the area under the curve was 0.985 and 0.825, respectively. Yingmei Fu et al. identified ASdes, a sRNA encoded by *M. tuberculosis*, raised, remarkably, in the plasma of active tuberculosis patients (Fu et al., 2018).

Small extracellular vesicles are nano-sized extracellular membrane vesicles (∼100 nm) that are secreted by almost all the eukaryotic cells (Hadifar et al., 2019). Mounting evidence has confirmed that small extracellular vesicles contain lots of biological molecules (e.g., proteins, lipids, and nucleic acids) and play an important role in many physiological and pathological processes (Hadifar et al., 2019). Exosomal RNAs have been considered as an ideal platform for the development of diagnostic biomarkers due to stability resulting from small extracellular vesicle-mediated protection against RNase degradation (Lv et al., 2017; Alipoor et al., 2019; Lyu et al., 2019; Liu et al., 2020). Previous reports indicated that small extracellular vesicles isolated from the *M. tuberculosis* infected macrophage contain *M. tuberculosis*-encoded sRNA and regulated host immunity (Singh et al., 2015; Cheng and Schorey, 2019; Layre, 2020). However, it is not known whether these *M. tuberculosis*-encoded sRNAs present in the small extracellular vesicles could serve as diagnostic biomarkers for *M. tuberculosis* infection. In this study, we chose *MTBmiR-5* and ASdes as examples to explore whether *M. tuberculosis-*encoded small RNA did exist in the small extracellular vesicles of serum and could serve as a diagnosis biomarker for active pulmonary tuberculosis.

## Materials and Methods

### Patients and Healthy Controls

Serum samples from 131 active pulmonary TB patients, 50 community-acquired pneumonia patients (CAP) and 50 healthy controls were collected at the patients’ first admission to the Nanjing Drum Tower Hospital. The active pulmonary TB patients were diagnosed by positive acid-fast bacilli (AFB) smear staining or sputum culture. The CAP patients were diagnosed by negative AFB smear staining and/or sputum culture but shared similar symptoms to active pulmonary TB infection. The healthy controls were collected from the physical examination center of Nanjing Drum Tower Hospital and had no clinical symptoms or negative AFB smear staining and/or sputum culture. Patients and controls were matched based on age and gender. The demographic and clinical characteristics of active TB patients, CAP patients, and healthy individuals were in Supplementary Table 1. Serum samples from 10 patients who had negative AFB smear staining and sputum culture at the first admission to the hospital but developed bacteriologically positive disease later during the hospital stay were also collected at the first admission to the hospital, and the second day after the result of AFB smear staining and/or the sputum culture changed positive. The protocol was approved by the Institutional Research Board of the Nanjing Drum Tower Hospital and performed according to the principles of the Helsinki Declaration.

### Serum Small Extracellular Vesicle Isolation and RNA Extraction

To establish the method to isolate the small extracellular vesicle from the serum by the Total Exosome Isolation Reagent (from serum) (Cat# 4478360, Invitrogen, United States) according to the manufacturer’s protocol, the isolated small extracellular vesicles were characterized by transmission electron microscopy (TEM), nanoparticle tracking analysis (NTA), and Western blotting as previously described (Jiao et al., 2020) (Zheng et al., 2021). Briefly, the TEM was performed by a JEM-1011 scanning transmission electron microscope (Hitachi, Tokyo, Japan). The ZetaView PMX 110 (Particle Metrix, Meerbusch, Germany) was used for nanoparticle tracking analysis according to the guidelines of the International Society for Extracellular Vesicles and the manufacturer’s protocol. The small extracellular vesicle protein markers CD63, CD81, and CD9 were detected by western blotting with CD63 (67605-1-Ig) and CD81 (66866-1-Ig) monoclonal antibodies (Proteintech, United States). To isolate the RNA from the small extracellular vesicles of serum for qRT-PCR, the small extracellular vesicle isolated method identified by TEM, NTA, and Western blotting was implemented. Briefly, a 100 μl serum sample was centrifuged at 2,000 × *g* for 30 min and 10,000 × *g* for 20 min to remove cells and debris. Immediately, the clarified serum was mixed with 0.2 volume of the total small extracellular vesicle isolation (from serum) reagent. The mixture was incubated at 2 to 8°C for 60 min. After incubation, the sample was centrifuged at 10,000 × *g* for 10 min at room temperature, and the supernatant was discarded. The small extracellular vesicles are contained in the pellet at the bottom of the tube and resuspended in a 100 μl phosphate-buffered saline.

### qRT-PCR

The total RNA from all the small extracellular vesicles of the 100 μl serum sample was isolated by the mirVana PARIS Kit (Ambion, Thermo Scientific, Shanghai, China). In order to normalize the qRT-PCR, the synthetic cel-miR-39 (5′-UCACCGGGUGUAAAUCAGCUUG-3′) (a *Caenorhabditis elegans*-encoded miRNA) (RiboBio, Guangzhou, China) was spiked into the denatured exosomes (Kroh et al., 2010). The total RNA was suspended in 20 μl of RNase-free water. Subsequently, the quantity and quality of total RNA was analyzed spectrophotometrically (NanoDrop 2,000, Thermo Scientific). For qRT-PCR, the SuperScript First-Strand Synthesis System (Invitrogen, Carlsbad, CA, United States) was used to synthesized the cDNA from the total RNA isolated from the small extracellular vesicle. The Roche LightCycler 96 was performed to implement the qRT-PCR reaction according to the manufacturer’s protocol. Briefly, reverse transcription reaction was performed for a total volume of 10 μl, including 3 μl of extracted RNA from each sample and 1 μl of RT primer at 16°C for 30 min, 42°C for 30 min, and 85°C for 5 min. Then, the PCR reactions were performed in a total volume of 10 μl, including 3 μl cDNA, 5 μl 2 × SYBR Green, 0.5 μl mixed primers at 95°C for 10 min, followed by 40 cycles of 95°C for 15 s and 60°C for 60 s (Jiao et al., 2020; Liu et al., 2020). All reactions, including the no-template controls, were analyzed in triplicate. The mean C_T_ value of each sample was determined from the triplicate reactions. To calculate the absolute expression levels of the *M. tuberculosis*-encoded small RNA, the two synthetic *M. tuberculosis*-encoded small RNA oligonucleotides at known concentrations in water were also reverse-transcribed and amplified. The quantity of each miRNA was then calculated by referring to the standard curve. The primers were synthesized at Genscript (Nanjing, China) (Supplementary Table 2). The raw CT value was presented in Supplementary Table 3.

### Expression and Statistical Analyses

The statistical analyses were performed by SPSS 18.0 software (SPSS Inc., Chicago, IL, United States). The graphs were generated by GraphPad Prism 6.0 (GraphPad Software, San Diego, CA, United States). To compare the differences in exosomal *M. tuberculosis*-encoded sRNA expression, the Mann-Whitney U test was used. A *p*-value < 0.05 was regarded as statistically significant. The diagnostic value of exosomal *M. tuberculosis*-encoded sRNA was estimated by Receiver Operating Characteristic (ROC) curve analysis.

## Results

### Characterization of Small Extracellular Vesicles

**FIGURE 1:**
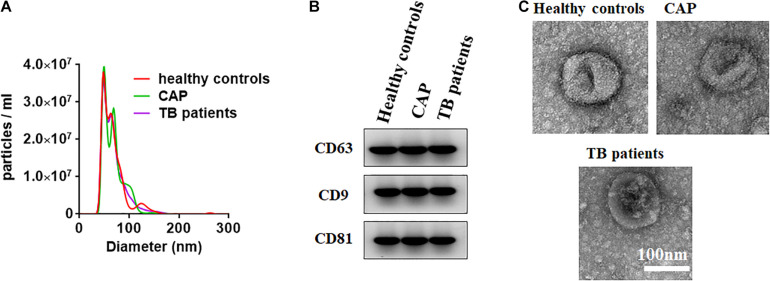
Characterization of small extracellular vesicles derived from the serum. **(A)** The size of small extracellular vesicles derived from serum of healthy controls, active pulmonary TB patients and community-acquired pneumonia (CAP) patients. **(B)** Western blots of exosomal membrane markers, including CD63, CD9, and CD81. **(C)** Shape and structure of serum exosomes isolated by ultracentrifugation under TEM.

The NTA, Western blotting, and TEM were performed to characterize the small extracellular vesicles derived from the serum isolated by the Total Exosome Isolation Reagent (from serum). As previously reported (Pegtel and Gould, 2019), the small extracellular vesicle diameters were found to be approximately 100 nm through nanoparticle tracking analysis (Figure 1A). As shown in Figure 1B, the small extracellular vesicle protein markers CD63, CD9, and CD81 was characterized by western blotting. We also investigated the EVs by TEM and found the typical size and rounded membrane-bound morphology of EVs isolated by the Total Exosome Isolation Reagent (from serum) (Figure 1C). Above all, the results from Western blotting and nanoparticle tracking analysis clearly confirmed that we had successfully isolated small extracellular vesicles from serum.

### Identify *Mycobacterium tuberculosis* Encoded sRNAs in the Small Extracellular Vesicles Derived From Serum

**FIGURE 2:**
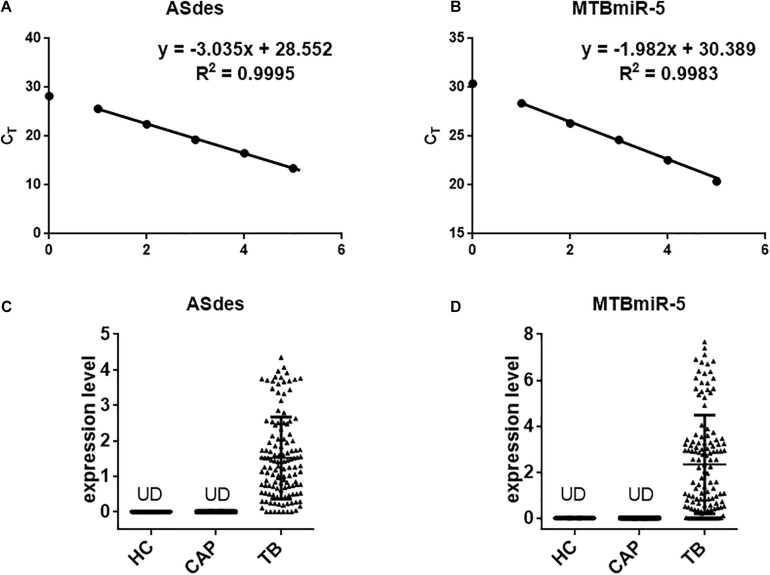
The changes in concentrations of serum exosomal *M. tuberculosis*-encoded ASdes and MTB-miR5. **(A,B)** Dynamic range and sensitivity of the qRT-PCR assay for measuring ASdes **(A)** and MTB-miR5 **(B)**. Synthetic single-stranded sRNA was serially diluted over several orders of magnitude to levels ranging from 10 attomole to 105 attomole and assessed by the qRT-PCR assay. The resulting CT values were plotted against the index with base 10 of the amount of input sRNA to generate a standard curve. An assay using water instead of RNA for the qRT-PCR assay was included as a no-template control. **(C,D)** The expression level (pmol per 1 L of serum) of ASdes **(C)** and MTB-miR5 **(D)** in the small extracellular vesicle isolated from serum of healthy controls (HC), active pulmonary TB patients (TB) and community acquired pneumonia (CAP) patients.

Bacterial small RNA (sRNA) is a class of regulatory RNA molecules. Several studies have identified *M. tuberculosis*-encoded sRNAs in the serum/plasma of *M. tuberculosis-*infected patients (Lv et al., 2017; Fu et al., 2018; Chakrabarty et al., 2019). Small extracellular vesicles are small membrane vesicles secreted by many cell types during physiological and pathological conditions (Hadifar et al., 2019). Recent evidence has indicated that most of the nucleic acids in the serum are packaged in the small extracellular vesicles and could serve as ideal diagnostic biomarkers (Hadifar et al., 2019). Previous reports indicated that small extracellular vesicles isolated from serum of TB patients contain mycobacterial proteins and DNA (Kruh-Garcia et al., 2014; Cho et al., 2020). In this study, we attempted a novel approach for TB diagnosis targeting exosomal sRNA by qRT-PCR. ASdes and MTB-miR5, two *M. tuberculosis*-encoded sRNAs (confirmed by Sanjiban Chakrabarty in 2019 (Chakrabarty et al., 2019) and Yingmei Fu in 2018 (Fu et al., 2018)), were chosen in this study (Table 1). To ensure the sRNA detection method is reliable, we firstly performed real-time PCR using synthesized ASdes and MTB-miR5, which were gradient-diluted as template. Using the formula for calculating the amplification efficiency, we found that the amplification efficiency of these two primer sets is all approximately equal to one (Figures 2A,B). The C_T_ value of the no-template controls for ASdes and MTB-miR5 was 28.04 and 30.08, respectively. These results indicated this real-time PCR detection method is specific and effective and could be used in further tests. Subsequently, ASdes and MTB-miR5 were identified in the small extracellular vesicles of serum isolated from the 131 active pulmonary TB patients, 50 community-acquired pneumonia (CAP) patients and 50 healthy controls by qRT-PCR. As shown in the results in Figures 2C,D, the ASdes and MTB-miR5 exist only in the small extracellular vesicles of serum isolated from active pulmonary TB patients but don’t present in the small extracellular vesicles of serum isolated from community acquired pneumonia and healthy controls. There were 114 (87%) (C_T_ < 30.08) positive results in the 131 active pulmonary TB patients of MTB-miR5 and 124 (94.7%) (C_T_ < 28.04) positive results in the 131 active pulmonary TB patients of ASdes.

**TABLE 1:** *M. tuberculosis*-encoded ASdes and MTB-miR5.

sRNA	Start	End	Sequence	Size
MTBmiR-5	1472988	1473009	UGAGAGACUGCCGGGGUCAACU	22
Asdes	918268	918365	AUAGAGGACGGAGUCGGUGAGG CUCUCCGCGAAAUAGUGGCCCU GGUGGUUUUGGCCUGGGCUGA AGCCCCGGUUGACUACCUCGAG GCGAAGUUUCU	98

### ROC Curve Analysis

**FIGURE 3:**
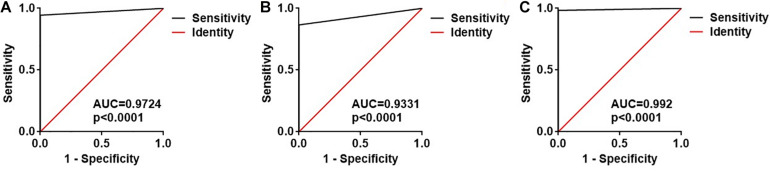
ROC curve analysis for the ASdes **(A)**, MTB-miR5 **(B)** and the combined two sRNA **(C)** panel in the small extracellular vesicle isolated from serum of healthy controls (HC), active pulmonary TB patients (TB), and community-acquired pneumonia (CAP) patients.

Subsequently, ROC curve analysis was performed to investigate the diagnostic value of the two exosomal *M. tuberculosis*-encoded sRNAs for *M. tuberculosis* infection. The areas under the curve (AUCs) of the ASdes and MTB-miR5 were 0.9724 (95% CI, 0.9491 to 0.9958) and 0.9331(95% CI, 0.8973 to 0.9688), respectively (Figures 3A,B). The sensitivity and specificity of ASdes were 94.49% and 100%, and the sensitivity and specificity of MTB-miR5 were 86.61% and 100%. In addition, the diagnostic values of the combinations of these two *M. tuberculosis*-encoded sRNAs were evaluated by a logistic regression model and found that the AUC was 0.9920 with *p* < 0.01 (95% CI, 0.9604 to 1.016) (Figure 3C). The sensitivity and specificity of the combinations of these two *M. tuberculosis* -encoded sRNAs was 97.62% and 100%. These results suggest that serum exosomal *M. tuberculosis*-encoded sRNAs have relatively high diagnostic accuracy for *M. tuberculosis* infection.

### Identify ASdes and MTB-miR5 in the Small Extracellular Vesicles Derived From Serum of TB Infection-Patients With Negative AFB Smear Staining and Sputum Culture at the First Admission to the Hospital

**FIGURE 4:**
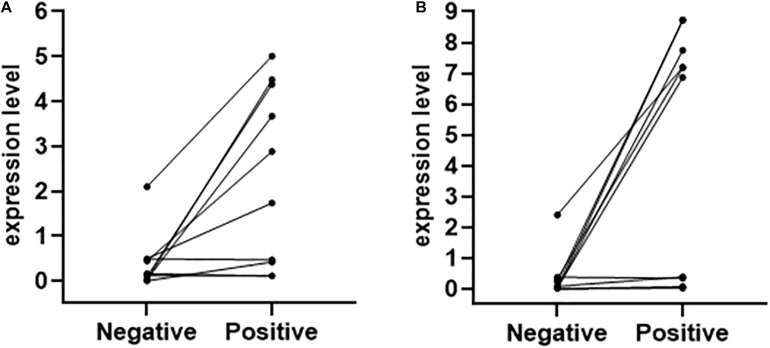
The expression level (pmol per 1L serum) of ASdes **(A)** and MTB-miR5 **(B)** in the small extracellular vesicles derived from serum of TB infection patients with negative AFB smear staining and sputum culture at the first admission to the hospital (negative) and the second day after the result of AFB smear staining or/and sputum culture changed positive (positive).

Diagnostic difficulties arise when AFB smear staining and sputum culture are negative for TB-infected patients with compatible symptoms and chest radiographs for tuberculosis. In order to explore the expression level of ASdes and MTB-miR5 in the small extracellular vesicles derived from the serum of TB infection-patients with negative AFB smear staining and sputum culture at the first admission to the hospital, we collected 10 serum samples that had negative AFB smear staining and sputum culture at the first admission to the hospital but developed bacteriologically positive disease during the hospital later. As shown in Figures 4A,B, nine of these 10 patients were positive for ASdes, while eight of the 10 patients were positive for MTB-miR5 (Table 2). These results suggest that serum exosomal *M. tuberculosis*-encoded ASdes and MTB-miR5 have the possibility to serve as ideal biomarker for TB infected-patients with negative AFB smear staining and sputum culture at the first admission to the hospital.

**TABLE 2:** The expression level (pmol per 1 L of serum) of ASdes and MTB-miR5 in the small extracellular vesicles derived from serum of TB-infected patients with negative AFB smear staining and sputum culture at the first admission to the hospital and the second day after the result of AFB smear staining or/and sputum culture changed positive.

	Negative		Positive	
	Asdes	MTBmiR-5	Asdes	MTBmiR-5
Patient 1	0.16	0.08	0.11	0.39
Patient 2	0.12	0.03	0.11	8.73
Patient 3	0.03	0.03	3.67	6.87
Patient 4	0.45	0.23	2.88	8.73
Patient 5	2.10	2.40	5.01	7.20
Patient 6	0.49	0.38	0.46	0.34
Patient 7	0.03	0.02	4.48	7.74
Patient 8	0.50	0.32	1.74	7.17
Patient 9	0.09	0.00	4.38	0.03
Patient 10	0.00	0.00	0.42	0.07

## Discussion

Tuberculosis remains one of the most important infectious diseases in people worldwide, with almost one-third of the world’s population infected by *M. tuberculosis* and one million dead each year (WHO, 2020). This is mainly due to the difficulty of diagnosis. Nowadays, the most commonly used diagnosis methods for *M. tuberculosis* are imaging inspection, bacteriological inspection, molecular biological detection, and immunological experimental inspection. However, almost all the diagnosis methods have disadvantages for *M. tuberculosis* detection. The specificity of typical imaging inspection and immunological experimental inspection is too low to distinguish the TB infection from other lung diseases, such as cancer and pneumonia. As the gold standard for TB diagnosis, the bacteriological inspection requires 4–8 weeks for the growth of *M. tuberculosis*. Moreover, the results of AFB smear staining and sputum culture were negative for many of the *M. tuberculosis*-infected patients. According to World Health Organization statistics, 40% of the *M. tuberculosis* patients failed to be identified and reported (WHO, 2020). There is an unmet clinical need for improved rapid and accurate diagnosis method for *M. tuberculosis* detection.

Small extracellular vesicles are membrane-bound vesicles released by all kinds of cells, and they play an important role in intercellular communication regulating various cellular functions of recipient cells. Small extracellular vesicles could protect RNA from hydrolysis by nuclease since they have a membrane structure. Thus, exosomal RNA has always been thought of as an ideal biomarker for disease diagnosis. Previous studies have found that *M. tuberculosis*-infected macrophages release small extracellular vesicles carrying *M. tuberculosis* transcripts, including small RNA. These small extracellular vesicle-carrying *M. tuberculosis* transcripts may be detected by pattern-recognition receptors on recipient cells to activate or attenuate cellular responses (Singh et al., 2015; Lv et al., 2017; Cheng and Schorey, 2019; Lyu et al., 2019; Layre, 2020). In our study, we attempted to evaluate the diagnostic value of the *M. tuberculosis-*encoded sRNA in the small extracellular vesicle for *M. tuberculosis* infection. First, the small extracellular vesicles were isolated from active pulmonary TB patients, CAP patients, and healthy controls, and then we identified two previously identified *M. tuberculosis-*encoded sRNA (ASdes and MTB-miR5) in these small extracellular vesicles. ASdes was identified in an *M. tuberculosis* culture supernatant and plasma of patients with active tuberculosis (Fu et al., 2018). Fu et al. (2018) found there were 15 (55.56%) positive results out of 27 active tuberculosis patients and 6 (25%) positive results in 24 healthy controls of ASdes. In our study, we improved the method to detect this *M. tuberculosis* encoded-sRNA by real-time qRT-PCR. The results showed the ASdes exist only in the small extracellular vesicles of serum isolated from active pulmonary TB patients but don’t appear in the small extracellular vesicles of serum isolated from community-acquired pneumonia and healthy controls. The ASdes could be detected in 124 active pulmonary TB patients of the 131 active pulmonary TB cases and the AUC was 0.9724. MTB-miR5 was confirmed by Sanjiban Chakrabarty (Chakrabarty et al., 2019) with small RNA sequencing and RT-PCR in the serum of *M. tuberculosis* infected patients. They found the AUC of MTBmiR-5 was about 0.985 with 76.92% sensitivity and 81.82% specificity (Chakrabarty et al., 2019). As for the ASdes, MTBmiR-5 was also only detected in the small extracellular vesicles of serum isolated from active pulmonary TB patients by real-time qRT-PCR in our study. The AUC of the MTB-miR5 was 0.9331 (95% CI, 0.8973 to 0.9688), and the sensitivity and specificity of MTB-miR5 were 86.61% and 100%. Moreover, we found the AUC of the combination of these two exosomal *M. tuberculosis-*encoded sRNA was 0.992, and the sensitivity and specificity were 97.62% and 100%. More interestingly, the ASdes and MTB-miR5 were positive in the *M. tuberculosis*-infected patients even through the results of AFB smear staining and sputum culture for these patients were negative. These results suggested the ASdes and MTB-miR5 in the small extracellular vesicle of serum may be considered as possible biomarkers for active TB.

There are some limitations to our study. First, we identified only two previously reported *M. tuberculosis-*encoded sRNA in the small extracellular vesicles. In order to comprehensively identify the sRNA encoded by *M. tuberculosis* in the small extracellular vesicles, it is necessary to use small RNA sequencing for the small extracellular vesicles isolated from the *M. tuberculosis-*infected patients. Some studies have shown the sRNA expression profiles of small extracellular vesicles isolated from *M. tuberculosis* infected macrophage or serum/plasma (Lv et al., 2017; Lyu et al., 2019). However, the sample size is too small for in-depth analysis. Secondly, although we confirmed *M. tuberculosis* encoded ASdes and MTB-miR5 could be packaged into small extracellular vesicles and serve as biomarkers for *M. tuberculosis* infection. However, the biological function of the exosomal ASdes and MTB-miR5 remains unknown. Several studies have suggested that *M. tuberculosis*-encoded sRNA could function as miRNA or long non-coding RNA in the host cells during infection (Masse et al., 2003; Doudna, 2011; Felden and Bouloc, 2017; Cheng and Schorey, 2019). Since the MTB-miR5 was 22 nt and derived from the hairpin structure (Fu et al., 2018), we speculate that it may play a miRNA-like role in the receipting cells. While the ASdes might act as a non-coding RNA since the size of ASdes was about 100 nt. However, it needs to be further confirmed *in vivo* and *in vitro*. Finally, the ASdes and MTB-miR5 in the small extracellular vesicles were just evaluated in about one hundred TB patients with one hundred controls. Further studies are needed in large multicenter longitudinal cohorts.

In summary, we have identified *M. tuberculosis*-encoded ASdes and MTB-miR5 in the small extracellular vesicles isolated from the serum of active pulmonary TB patients. These two exosomal *M. tuberculosis*-encoded sRNA has the potential to be a minimally invasive diagnostic screening tool for *M. tuberculosis* infection.

## Data Availability Statement

The original contributions presented in the study are included in the article/Supplementary Material, further inquiries can be directed to the corresponding author/s.

## Ethics Statement

The studies involving human participants were reviewed and approved by Medical Ethics Committee, Nanjing Drum Tower Hospital, and The Affiliated Hospital of Nanjing University Medical School. The patients/participants provided their written informed consent to participate in this study.

## Author Contributions

JW and SG designed the experiments. GL, XJ, JZ, FH, QZ, and AW performed the experiments and analyzed the results. JW wrote the manuscript. KZ provided a critical reading of the manuscript. All authors contributed to the article and approved the submitted version.

## Conflict of Interest

The authors declare that the research was conducted in the absence of any commercial or financial relationships that could be construed as a potential conflict of interest.
