# Supplementary material for: Two Small Extracellular Vesicle sRNAs Derived From Mycobacterium tuberculosis Serve as Diagnostic Biomarkers for Active Pulmonary Tuberculosis
Source: Front Microbiol. 2021 Apr 15;12:642559. doi: 10.3389/fmicb.2021.642559 (PMC8082067; doi:10.3389/fmicb.2021.642559)
Supplement: Supplementary file 1 [file Table_1.DOCX]

**Supplement materials**

**Table s1. The clinical characteristics of healthy controls, active pulmonary TB patients and community acquired pneumonia (CAP) patients.**

|  | **TB (N=131)** | **CAP (N=50)** | **HC (N=50)** |
| --- | --- | --- | --- |
| Age,years^a^ | 42.7(20.05) | 40.8(10.45) | 40.5(16.65) |
| Sex,n |  |  |  |
| Male | 89 | 30 | 22 |
| Female | 42 | 20 | 28 |

**Table s2. Primers used for detection of sRNA.**

|  | **Primer(5'-3')** | |
| --- | --- | --- |
| **ASdes** | **RT** | CTTCGCCTCGAGGTAGTCAACC |
|  | **F** | ATAGAGGACGGAGTCGGTGA |
|  | **R** | AGAAACTTCGCCTCGAGGTA |
|  | **Probe** | GAAATAGTGGCCC |
| **MTBmiR-5** | **RT** | GTCGTATCCAGTGCAGGGTCCGAGGTATTCGCACTGGATACGACAGTTGA |
|  | **F** | GTCGTATCCAGTGCAGGGT |
|  | **R** | AACAAGTGAGAGACTGCCGG |
|  | **Probe** | GTCAACTGTCGTAT |

**Table s3. The raw C_T_ of the qRT-PCR.**

| **concentration (amol/ul)** | **cel-miR-39#1** | **cel-miR-39#2** | **cel-miR-39#3** | **Asdes#1** | **Asdes#2** | **Asdes#3** | **MTBmiR-5#1** | **MTBmiR-5#2** | **MTBmiR-5#3** |
| --- | --- | --- | --- | --- | --- | --- | --- | --- | --- |
| **No-Template Control** | 29.72 | 29.46 | 29.62 | 28.08 | 28.03 | 28.02 | 29.92 | 30.18 | 30.15 |
| **10.00** | 27.59 | 27.61 | 27.12 | 25.41 | 25.24 | 25.11 | 28.49 | 28.44 | 28.31 |
| **100.00** | 25.35 | 25.68 | 25.15 | 21.89 | 21.99 | 21.96 | 26.34 | 26.53 | 26.43 |
| **1000.00** | 23.11 | 23.21 | 23.16 | 18.72 | 18.44 | 18.76 | 24.41 | 24.51 | 24.39 |
| **10000.00** | 21.18 | 21.14 | 21.59 | 15.14 | 15.56 | 15.33 | 22.39 | 22.38 | 22.62 |
| **100000.00** | 19.70 | 19.96 | 19.89 | 12.03 | 12.01 | 12.05 | 20.43 | 20.64 | 20.37 |

|  | **cel-miR-39** | | | **ASdes** | | | **MTBmiR-5** | | |
| --- | --- | --- | --- | --- | --- | --- | --- | --- | --- |
| **no.** | **HC#1** | **HC#2** | **HC#3** | **HC#1** | **HC#2** | **HC#3** | **HC#1** | **HC#2** | **HC#3** |
| **HC1** | 24.06 | 24.11 | 24.99 | 28.15 | 28.74 | 28.77 | 32.19 | 33.17 | 33.13 |
| **HC2** | 24.92 | 24.91 | 24.99 | 28.15 | 28.79 | 28.77 | 32.19 | 33.08 | 33.13 |
| **HC3** | 24.16 | 24.11 | 24.98 | 28.22 | 28.81 | 28.81 | 32.69 | 33.06 | 33.04 |
| **HC4** | 24.32 | 24.30 | 24.96 | 28.22 | 28.80 | 28.81 | 32.69 | 33.01 | 33.04 |
| **HC5** | 24.76 | 24.79 | 24.93 | 28.21 | 28.28 | 28.27 | 32.02 | 32.99 | 32.98 |
| **HC6** | 24.72 | 24.70 | 24.89 | 28.21 | 28.26 | 28.27 | 32.02 | 32.96 | 32.98 |
| **HC7** | 24.90 | 24.91 | 24.87 | 28.99 | 28.61 | 28.60 | 32.78 | 32.08 | 32.08 |
| **HC8** | 24.81 | 24.79 | 24.86 | 28.99 | 28.58 | 28.60 | 32.78 | 32.08 | 32.08 |
| **HC9** | 24.40 | 24.38 | 24.82 | 28.11 | 28.65 | 28.70 | 30.95 | 31.93 | 31.92 |
| **HC10** | 24.76 | 24.79 | 24.82 | 28.11 | 28.74 | 28.70 | 30.95 | 31.90 | 31.92 |
| **HC11** | 24.35 | 24.38 | 24.80 | 28.96 | 28.12 | 28.11 | 32.79 | 31.61 | 31.61 |
| **HC12** | 24.42 | 24.38 | 24.80 | 28.96 | 28.09 | 28.11 | 32.79 | 31.61 | 31.61 |
| **HC13** | 24.34 | 24.38 | 24.77 | 28.79 | 28.14 | 28.18 | 32.81 | 31.58 | 31.57 |
| **HC14** | 24.14 | 24.09 | 24.75 | 28.79 | 28.21 | 28.18 | 32.81 | 31.55 | 31.57 |
| **HC15** | 24.14 | 24.15 | 24.62 | 28.09 | 29.60 | 29.63 | 31.66 | 31.51 | 31.50 |
| **HC16** | 24.04 | 24.09 | 24.60 | 28.09 | 29.66 | 29.63 | 31.66 | 31.49 | 31.50 |
| **HC17** | 24.79 | 24.79 | 24.59 | 29.00 | 28.20 | 28.21 | 32.28 | 31.50 | 31.46 |
| **HC18** | 24.45 | 24.44 | 24.57 | 29.00 | 28.21 | 28.21 | 32.28 | 31.41 | 31.46 |
| **HC19** | 24.79 | 24.79 | 24.57 | 28.56 | 28.24 | 28.25 | 32.22 | 31.37 | 31.35 |
| **HC20** | 24.43 | 24.44 | 24.54 | 28.56 | 28.25 | 28.25 | 32.22 | 31.32 | 31.35 |
| **HC21** | 24.16 | 24.15 | 24.53 | 28.38 | 28.94 | 28.97 | 31.73 | 31.06 | 31.03 |
| **HC22** | 24.46 | 24.48 | 24.48 | 28.38 | 28.99 | 28.97 | 31.73 | 31.00 | 31.03 |
| **HC23** | 24.49 | 24.48 | 24.45 | 28.84 | 28.44 | 28.45 | 31.49 | 30.97 | 30.97 |
| **HC24** | 24.89 | 24.91 | 24.38 | 28.84 | 28.45 | 28.45 | 31.49 | 30.97 | 30.97 |
| **HC25** | 24.92 | 24.91 | 24.38 | 28.58 | 28.87 | 28.87 | 31.79 | 32.82 | 32.79 |
| **HC26** | 24.21 | 24.18 | 24.34 | 28.58 | 28.86 | 28.87 | 31.79 | 32.76 | 32.79 |
| **HC27** | 24.05 | 24.00 | 24.33 | 28.93 | 28.95 | 29.03 | 33.71 | 32.64 | 32.64 |
| **HC28** | 24.15 | 24.18 | 24.33 | 28.93 | 28.10 | 28.03 | 33.71 | 32.63 | 32.64 |
| **HC29** | 24.20 | 24.20 | 24.32 | 29.00 | 28.82 | 28.93 | 31.14 | 32.57 | 32.54 |
| **HC30** | 24.19 | 24.20 | 24.30 | 29.00 | 28.03 | 27.93 | 31.14 | 32.51 | 32.54 |
| **HC31** | 24.26 | 24.37 | 24.29 | 28.58 | 28.10 | 28.09 | 31.80 | 32.47 | 32.47 |
| **HC32** | 24.48 | 24.37 | 24.28 | 28.58 | 28.07 | 28.09 | 31.80 | 32.47 | 32.47 |
| **HC33** | 24.53 | 24.53 | 24.24 | 28.29 | 28.54 | 28.59 | 33.44 | 32.22 | 32.20 |
| **HC34** | 24.66 | 24.59 | 24.23 | 28.29 | 28.63 | 28.59 | 33.44 | 32.18 | 32.20 |
| **HC35** | 24.35 | 24.35 | 24.22 | 28.17 | 28.46 | 28.47 | 31.94 | 32.19 | 32.16 |
| **HC36** | 24.52 | 24.59 | 24.20 | 28.17 | 28.48 | 28.47 | 31.94 | 32.13 | 32.16 |
| **HC37** | 24.34 | 24.35 | 24.19 | 28.34 | 28.01 | 28.03 | 31.31 | 32.04 | 32.01 |
| **HC38** | 24.27 | 24.27 | 24.18 | 28.34 | 28.05 | 28.03 | 31.31 | 31.97 | 32.01 |
| **HC39** | 24.69 | 24.72 | 24.17 | 28.31 | 28.81 | 28.60 | 33.17 | 31.95 | 31.92 |
| **HC40** | 24.26 | 24.27 | 24.16 | 28.31 | 28.76 | 28.60 | 31.83 | 31.92 | 31.92 |
| **HC41** | 24.31 | 24.24 | 24.14 | 28.47 | 28.38 | 28.60 | 31.83 | 31.91 | 31.92 |
| **HC42** | 24.74 | 24.72 | 24.13 | 28.47 | 28.43 | 28.60 | 33.17 | 31.89 | 31.92 |
| **HC43** | 24.16 | 24.18 | 24.11 | 28.81 | 28.41 | 28.39 | 31.04 | 31.79 | 31.79 |
| **HC44** | 24.20 | 24.18 | 24.11 | 28.81 | 28.37 | 28.39 | 31.04 | 31.79 | 31.79 |
| **HC45** | 24.82 | 24.79 | 24.07 | 28.13 | 28.10 | 28.12 | 31.38 | 31.64 | 31.51 |
| **HC46** | 24.16 | 24.24 | 24.04 | 28.13 | 28.14 | 28.12 | 31.38 | 31.59 | 31.51 |
| **HC47** | 24.28 | 24.30 | 24.04 | 28.19 | 28.02 | 28.06 | 32.77 | 31.43 | 31.51 |
| **HC48** | 24.95 | 25.00 | 24.04 | 28.19 | 28.09 | 28.06 | 32.77 | 31.38 | 31.51 |
| **HC49** | 24.67 | 24.70 | 24.02 | 28.68 | 28.97 | 28.98 | 32.38 | 31.85 | 31.84 |
| **HC50** | 24.52 | 24.53 | 24.01 | 28.68 | 28.98 | 28.98 | 32.38 | 31.82 | 31.84 |

|  | **cel-miR-39** | | | **ASdes** | | | **MTBmiR-5** | | |
| --- | --- | --- | --- | --- | --- | --- | --- | --- | --- |
| **no.** | **CAP#1** | **CAP#2** | **CAP#3** | **CAP#1** | **CAP#2** | **CAP#3** | **CAP#1** | **CAP#2** | **CAP#3** |
| **CAP1** | 24.74 | 24.75 | 24.98 | 28.45 | 28.80 | 28.82 | 32.30 | 30.93 | 30.87 |
| **CAP2** | 24.76 | 24.78 | 24.97 | 28.45 | 28.83 | 28.82 | 32.30 | 30.80 | 30.87 |
| **CAP3** | 24.05 | 24.05 | 24.95 | 28.69 | 28.54 | 28.57 | 31.04 | 30.68 | 30.67 |
| **CAP4** | 24.75 | 24.75 | 24.95 | 28.69 | 28.59 | 28.57 | 31.04 | 30.65 | 30.67 |
| **CAP5** | 24.04 | 24.05 | 24.92 | 28.65 | 28.44 | 28.45 | 31.77 | 30.72 | 30.64 |
| **CAP6** | 24.37 | 24.32 | 24.90 | 28.65 | 28.46 | 28.45 | 31.77 | 30.55 | 30.64 |
| **CAP7** | 24.93 | 24.96 | 24.89 | 28.28 | 28.10 | 28.16 | 30.45 | 30.61 | 30.60 |
| **CAP8** | 24.26 | 24.32 | 24.88 | 28.28 | 28.22 | 28.16 | 30.45 | 30.58 | 30.60 |
| **CAP9** | 24.98 | 24.96 | 24.87 | 28.78 | 28.27 | 28.27 | 30.98 | 30.60 | 30.59 |
| **CAP10** | 24.37 | 24.41 | 24.83 | 28.78 | 30.10 | 30.11 | 30.98 | 30.57 | 30.59 |
| **CAP11** | 24.16 | 24.19 | 24.80 | 28.97 | 28.58 | 28.60 | 33.63 | 30.35 | 30.34 |
| **CAP12** | 24.69 | 24.65 | 24.79 | 28.97 | 28.61 | 28.60 | 33.63 | 30.33 | 30.34 |
| **CAP13** | 24.83 | 24.81 | 24.77 | 28.99 | 28.15 | 28.15 | 31.80 | 30.33 | 30.31 |
| **CAP14** | 24.60 | 24.65 | 24.76 | 28.99 | 28.15 | 28.15 | 31.80 | 30.28 | 30.31 |
| **CAP15** | 24.37 | 24.38 | 24.73 | 28.23 | 29.29 | 29.29 | 31.65 | 32.11 | 32.10 |
| **CAP16** | 24.79 | 24.81 | 24.72 | 28.23 | 29.29 | 29.29 | 31.65 | 32.09 | 32.10 |
| **CAP17** | 24.38 | 24.40 | 24.71 | 28.94 | 28.78 | 28.79 | 30.57 | 32.10 | 32.07 |
| **CAP18** | 24.39 | 24.38 | 24.71 | 28.94 | 28.79 | 28.79 | 30.57 | 32.03 | 32.07 |
| **CAP19** | 24.58 | 24.59 | 24.70 | 28.89 | 29.13 | 29.18 | 30.48 | 31.97 | 31.96 |
| **CAP20** | 24.57 | 24.50 | 24.69 | 28.89 | 29.22 | 29.18 | 30.48 | 31.95 | 31.96 |
| **CAP21** | 24.42 | 24.50 | 24.68 | 28.49 | 28.44 | 28.44 | 32.11 | 31.95 | 31.93 |
| **CAP22** | 24.06 | 24.14 | 24.66 | 28.49 | 28.43 | 28.44 | 32.11 | 31.90 | 31.93 |
| **CAP23** | 24.22 | 24.14 | 24.65 | 28.75 | 28.85 | 28.92 | 30.85 | 31.78 | 31.76 |
| **CAP24** | 24.22 | 24.19 | 24.64 | 28.75 | 28.98 | 28.92 | 30.85 | 31.74 | 31.76 |
| **CAP25** | 24.59 | 24.59 | 24.62 | 28.88 | 28.64 | 28.65 | 32.19 | 31.69 | 31.66 |
| **CAP26** | 24.41 | 24.40 | 24.58 | 28.88 | 28.65 | 28.65 | 32.19 | 31.63 | 31.66 |
| **CAP27** | 24.95 | 24.91 | 24.53 | 28.66 | 28.51 | 28.51 | 31.99 | 31.66 | 31.60 |
| **CAP28** | 24.30 | 24.33 | 24.52 | 28.66 | 28.51 | 28.51 | 31.99 | 31.54 | 31.60 |
| **CAP29** | 24.87 | 24.91 | 24.52 | 28.48 | 28.54 | 28.52 | 31.38 | 31.58 | 31.53 |
| **CAP30** | 24.54 | 24.51 | 24.49 | 28.48 | 28.50 | 28.52 | 31.38 | 31.47 | 31.53 |
| **CAP31** | 24.36 | 24.33 | 24.46 | 28.53 | 28.06 | 28.05 | 30.46 | 31.42 | 31.33 |
| **CAP32** | 24.47 | 24.51 | 24.46 | 28.53 | 28.03 | 28.05 | 30.46 | 31.24 | 31.33 |
| **CAP33** | 24.57 | 24.57 | 24.46 | 28.66 | 29.51 | 29.48 | 30.52 | 31.35 | 31.32 |
| **CAP34** | 24.57 | 24.57 | 24.44 | 28.66 | 29.45 | 29.48 | 32.05 | 31.32 | 31.32 |
| **CAP35** | 24.54 | 24.55 | 24.40 | 28.27 | 28.68 | 28.68 | 32.05 | 31.31 | 31.32 |
| **CAP36** | 24.60 | 24.55 | 24.37 | 28.27 | 28.67 | 28.68 | 30.52 | 31.28 | 31.32 |
| **CAP37** | 24.56 | 24.55 | 24.34 | 28.09 | 28.02 | 28.05 | 31.76 | 31.36 | 31.31 |
| **CAP38** | 24.92 | 24.91 | 24.32 | 28.09 | 28.07 | 28.05 | 31.76 | 31.25 | 31.31 |
| **CAP39** | 24.03 | 24.10 | 24.31 | 28.72 | 28.02 | 28.05 | 31.63 | 31.14 | 31.14 |
| **CAP40** | 24.50 | 24.55 | 24.30 | 28.72 | 28.08 | 28.05 | 31.63 | 31.13 | 31.14 |
| **CAP41** | 24.30 | 24.30 | 24.27 | 28.92 | 28.47 | 28.49 | 32.06 | 31.06 | 31.01 |
| **CAP42** | 24.89 | 24.91 | 24.24 | 28.92 | 28.50 | 28.49 | 31.25 | 31.01 | 31.01 |
| **CAP43** | 24.16 | 24.10 | 24.23 | 28.37 | 28.58 | 28.63 | 31.25 | 31.00 | 31.01 |
| **CAP44** | 24.26 | 24.22 | 24.18 | 28.37 | 28.67 | 28.63 | 32.06 | 30.95 | 31.01 |
| **CAP45** | 24.82 | 24.76 | 24.16 | 28.14 | 28.15 | 28.19 | 30.62 | 31.12 | 32.07 |
| **CAP46** | 24.65 | 24.63 | 24.12 | 28.14 | 28.22 | 28.19 | 30.62 | 31.02 | 32.07 |
| **CAP47** | 24.70 | 24.76 | 24.11 | 28.34 | 28.43 | 28.46 | 31.56 | 30.89 | 31.86 |
| **CAP48** | 24.61 | 24.63 | 24.06 | 28.34 | 28.49 | 28.46 | 31.56 | 30.83 | 31.86 |
| **CAP49** | 24.45 | 24.41 | 24.01 | 28.72 | 28.15 | 28.17 | 31.48 | 31.03 | 31.02 |
| **CAP50** | 24.79 | 24.78 | 24.00 | 28.72 | 28.18 | 28.17 | 31.48 | 31.00 | 31.02 |

|  | **cel-miR-39** | | | **ASdes** | | | **MTBmiR-5** | | |
| --- | --- | --- | --- | --- | --- | --- | --- | --- | --- |
| **no.** | **TB#1** | **TB#2** | **TB#3** | **TB#1** | **TB#2** | **TB#3** | **TB#1** | **TB#2** | **TB#3** |
| **TB1** | 24.06 | 24.06 | 24.99 | 20.50 | 20.52 | 20.48 | 24.70 | 24.71 | 24.70 |
| **TB2** | 24.17 | 24.22 | 24.93 | 20.78 | 20.81 | 20.76 | 24.86 | 24.87 | 24.86 |
| **TB3** | 24.12 | 23.99 | 24.22 | 23.98 | 24.01 | 23.96 | 28.71 | 28.73 | 28.71 |
| **TB4** | 24.16 | 24.18 | 24.20 | 23.94 | 23.97 | 23.92 | 28.71 | 28.73 | 28.71 |
| **TB5** | 24.26 | 24.22 | 24.18 | 23.98 | 24.01 | 23.96 | 28.71 | 28.73 | 28.71 |
| **TB6** | 24.13 | 24.13 | 24.17 | 24.03 | 24.05 | 24.00 | 28.71 | 28.73 | 28.71 |
| **TB7** | 24.84 | 24.87 | 24.17 | 23.98 | 24.01 | 23.96 | 28.71 | 28.73 | 28.71 |
| **TB8** | 24.39 | 24.40 | 24.16 | 24.31 | 24.33 | 24.29 | 29.60 | 29.61 | 29.60 |
| **TB9** | 24.28 | 24.24 | 24.15 | 24.31 | 24.33 | 24.29 | 28.60 | 28.61 | 28.60 |
| **TB10** | 24.59 | 24.58 | 24.15 | 24.36 | 24.39 | 24.34 | 29.60 | 29.61 | 29.60 |
| **TB11** | 24.60 | 24.59 | 24.15 | 25.84 | 25.76 | 25.27 | 30.14 | 30.91 | 30.34 |
| **TB12** | 24.58 | 24.57 | 24.14 | 26.00 | 26.42 | 26.35 | 30.89 | 30.32 | 30.34 |
| **TB13** | 24.60 | 24.57 | 24.93 | 20.79 | 20.81 | 20.76 | 24.88 | 24.89 | 24.88 |
| **TB14** | 24.76 | 24.78 | 24.14 | 25.56 | 25.98 | 25.92 | 30.32 | 30.49 | 30.96 |
| **TB15** | 24.83 | 24.84 | 24.14 | 25.58 | 25.51 | 25.86 | 30.91 | 30.89 | 30.08 |
| **TB16** | 24.56 | 24.57 | 24.13 | 25.38 | 25.01 | 25.84 | 30.49 | 30.14 | 30.53 |
| **TB17** | 24.12 | 24.10 | 24.11 | 26.09 | 26.79 | 26.82 | 30.59 | 30.89 | 30.22 |
| **TB18** | 24.19 | 24.18 | 24.11 | 26.79 | 26.89 | 26.94 | 30.16 | 30.59 | 30.15 |
| **TB19** | 24.37 | 24.34 | 24.11 | 27.96 | 27.30 | 27.91 | 30.15 | 30.97 | 30.77 |
| **TB20** | 24.77 | 24.76 | 24.10 | 27.11 | 27.97 | 27.32 | 30.12 | 30.33 | 30.97 |
| **TB21** | 24.08 | 24.10 | 24.09 | 27.99 | 27.81 | 27.41 | 30.62 | 30.89 | 30.33 |
| **TB22** | 24.81 | 24.84 | 24.09 | 28.38 | 28.08 | 28.43 | 30.46 | 30.89 | 30.66 |
| **TB23** | 24.84 | 24.84 | 24.08 | 28.00 | 28.16 | 28.11 | 30.68 | 30.78 | 30.43 |
| **TB24** | 24.71 | 24.70 | 24.91 | 20.78 | 20.80 | 20.75 | 24.88 | 24.89 | 24.88 |
| **TB25** | 24.90 | 24.91 | 24.08 | 28.09 | 28.83 | 28.35 | 30.21 | 30.33 | 30.81 |
| **TB26** | 24.54 | 24.57 | 24.07 | 28.21 | 28.10 | 28.44 | 30.38 | 30.75 | 30.40 |
| **TB27** | 24.91 | 24.91 | 24.07 | 28.22 | 28.83 | 28.08 | 30.96 | 30.44 | 30.58 |
| **TB28** | 24.02 | 24.02 | 24.06 | 28.15 | 28.08 | 28.57 | 30.14 | 30.35 | 30.47 |
| **TB29** | 24.32 | 24.34 | 24.06 | 28.53 | 28.11 | 28.68 | 30.61 | 30.40 | 30.69 |
| **TB30** | 24.55 | 24.54 | 24.06 | 21.26 | 21.28 | 21.24 | 29.00 | 29.02 | 29.00 |
| **TB31** | 24.40 | 24.41 | 24.05 | 21.55 | 21.57 | 21.53 | 29.00 | 29.02 | 29.00 |
| **TB32** | 24.36 | 24.34 | 24.04 | 22.00 | 22.02 | 21.98 | 29.00 | 29.02 | 29.00 |
| **TB33** | 24.65 | 24.63 | 24.04 | 21.99 | 22.02 | 21.97 | 29.00 | 29.02 | 29.00 |
| **TB34** | 24.68 | 24.70 | 24.04 | 22.01 | 22.03 | 21.99 | 29.00 | 29.02 | 29.00 |
| **TB35** | 24.37 | 24.31 | 24.90 | 20.78 | 20.80 | 20.76 | 24.91 | 24.92 | 24.91 |
| **TB36** | 24.41 | 24.41 | 24.02 | 22.14 | 22.16 | 22.11 | 29.00 | 29.02 | 29.00 |
| **TB37** | 24.62 | 24.63 | 24.02 | 21.90 | 21.93 | 21.88 | 29.00 | 29.02 | 29.00 |
| **TB38** | 24.38 | 24.43 | 24.90 | 20.83 | 20.86 | 20.81 | 24.91 | 24.93 | 24.92 |
| **TB39** | 24.90 | 24.94 | 24.90 | 20.86 | 20.88 | 20.84 | 24.94 | 24.95 | 24.94 |
| **TB40** | 24.11 | 24.11 | 24.89 | 20.87 | 20.89 | 20.84 | 24.95 | 24.96 | 24.95 |
| **TB41** | 24.89 | 24.94 | 24.89 | 20.90 | 20.92 | 20.88 | 24.96 | 24.97 | 24.96 |
| **TB42** | 24.10 | 24.11 | 24.88 | 20.90 | 20.93 | 20.88 | 24.97 | 24.99 | 24.98 |
| **TB43** | 24.75 | 24.80 | 24.87 | 20.99 | 21.01 | 20.96 | 24.98 | 24.99 | 24.98 |
| **TB44** | 24.18 | 24.17 | 24.99 | 20.62 | 20.65 | 20.60 | 24.72 | 24.73 | 24.72 |
| **TB45** | 24.78 | 24.77 | 24.87 | 21.12 | 21.14 | 21.10 | 24.99 | 25.00 | 24.99 |
| **TB46** | 24.98 | 24.94 | 24.87 | 21.22 | 21.25 | 21.20 | 25.01 | 25.02 | 25.01 |
| **TB47** | 24.60 | 24.63 | 24.86 | 21.26 | 21.29 | 21.24 | 25.07 | 25.09 | 25.07 |
| **TB48** | 24.87 | 24.85 | 24.86 | 21.30 | 21.32 | 21.28 | 25.23 | 25.24 | 25.23 |
| **TB49** | 24.60 | 24.56 | 24.85 | 21.28 | 21.30 | 21.26 | 25.28 | 25.29 | 25.28 |
| **TB50** | 24.71 | 24.73 | 24.84 | 21.32 | 21.34 | 21.29 | 25.32 | 25.33 | 25.32 |
| **TB51** | 24.63 | 24.61 | 24.84 | 21.33 | 21.36 | 21.31 | 25.34 | 25.35 | 25.34 |
| **TB52** | 24.46 | 24.49 | 24.82 | 21.34 | 21.37 | 21.32 | 25.35 | 25.36 | 25.35 |
| **TB53** | 24.48 | 24.45 | 24.82 | 21.32 | 21.35 | 21.30 | 25.37 | 25.38 | 25.37 |
| **TB54** | 24.52 | 24.49 | 24.82 | 21.40 | 21.42 | 21.37 | 25.38 | 25.39 | 25.38 |
| **TB55** | 24.41 | 24.40 | 24.98 | 20.68 | 20.71 | 20.66 | 24.75 | 24.76 | 24.75 |
| **TB56** | 24.39 | 24.36 | 24.81 | 21.46 | 21.48 | 21.44 | 25.38 | 25.40 | 25.38 |
| **TB57** | 24.52 | 24.56 | 24.81 | 21.50 | 21.53 | 21.48 | 25.39 | 25.40 | 25.39 |
| **TB58** | 24.58 | 24.61 | 24.81 | 21.55 | 21.57 | 21.53 | 25.38 | 25.39 | 25.38 |
| **TB59** | 24.08 | 24.05 | 24.79 | 21.56 | 21.59 | 21.54 | 25.42 | 25.44 | 25.42 |
| **TB60** | 24.63 | 24.63 | 24.78 | 21.57 | 21.60 | 21.55 | 25.42 | 25.43 | 25.42 |
| **TB61** | 24.13 | 24.08 | 24.77 | 21.58 | 21.61 | 21.56 | 25.45 | 25.46 | 25.45 |
| **TB62** | 24.75 | 24.77 | 24.74 | 21.64 | 21.66 | 21.61 | 25.43 | 25.45 | 25.43 |
| **TB63** | 24.33 | 24.36 | 24.73 | 21.67 | 21.69 | 21.65 | 25.43 | 25.44 | 25.43 |
| **TB64** | 24.99 | 24.94 | 24.72 | 21.67 | 21.69 | 21.65 | 25.44 | 25.45 | 25.44 |
| **TB65** | 24.68 | 24.70 | 24.70 | 21.71 | 21.74 | 21.69 | 25.48 | 25.49 | 25.48 |
| **TB66** | 24.57 | 24.59 | 24.97 | 20.71 | 20.74 | 20.69 | 24.77 | 24.79 | 24.78 |
| **TB67** | 24.47 | 24.43 | 24.68 | 21.78 | 21.81 | 21.76 | 25.51 | 25.52 | 25.51 |
| **TB68** | 24.25 | 24.31 | 24.66 | 21.82 | 21.85 | 21.80 | 25.51 | 25.52 | 25.51 |
| **TB69** | 24.91 | 24.88 | 24.66 | 21.80 | 21.82 | 21.78 | 25.51 | 25.52 | 25.51 |
| **TB70** | 24.72 | 24.70 | 24.65 | 21.81 | 21.84 | 21.79 | 25.54 | 25.55 | 25.54 |
| **TB71** | 24.82 | 24.85 | 24.63 | 21.82 | 21.85 | 21.80 | 25.53 | 25.54 | 25.53 |
| **TB72** | 24.46 | 24.47 | 24.62 | 21.87 | 21.89 | 21.85 | 25.53 | 25.54 | 25.53 |
| **TB73** | 24.03 | 24.08 | 24.61 | 21.95 | 21.97 | 21.92 | 25.54 | 25.55 | 25.54 |
| **TB74** | 24.76 | 24.68 | 24.60 | 21.95 | 21.97 | 21.92 | 25.57 | 25.58 | 25.57 |
| **TB75** | 24.48 | 24.47 | 24.59 | 21.93 | 21.95 | 21.91 | 25.56 | 25.58 | 25.56 |
| **TB76** | 24.88 | 24.90 | 24.59 | 21.90 | 21.93 | 21.88 | 25.57 | 25.58 | 25.57 |
| **TB77** | 24.86 | 24.99 | 24.97 | 20.73 | 20.75 | 20.70 | 24.79 | 24.81 | 24.79 |
| **TB78** | 24.56 | 24.58 | 24.58 | 21.90 | 21.93 | 21.88 | 25.59 | 25.60 | 25.59 |
| **TB79** | 24.92 | 24.90 | 24.56 | 21.90 | 21.93 | 21.88 | 25.65 | 25.67 | 25.65 |
| **TB80** | 24.55 | 24.56 | 24.55 | 22.02 | 22.04 | 22.00 | 25.65 | 25.66 | 25.65 |
| **TB81** | 24.74 | 24.73 | 24.55 | 21.98 | 22.01 | 21.96 | 25.66 | 25.67 | 25.66 |
| **TB82** | 24.18 | 24.17 | 24.54 | 21.97 | 22.00 | 21.95 | 25.66 | 25.68 | 25.66 |
| **TB83** | 24.85 | 24.88 | 24.54 | 22.01 | 22.03 | 21.99 | 25.79 | 25.80 | 25.79 |
| **TB84** | 24.02 | 24.05 | 24.52 | 22.04 | 22.06 | 22.02 | 25.78 | 25.79 | 25.78 |
| **TB85** | 24.91 | 24.90 | 24.52 | 22.01 | 22.03 | 21.99 | 25.90 | 25.91 | 25.90 |
| **TB86** | 24.57 | 24.56 | 24.50 | 22.03 | 22.05 | 22.01 | 25.97 | 25.98 | 25.97 |
| **TB87** | 24.76 | 24.78 | 24.49 | 21.98 | 22.01 | 21.96 | 26.07 | 26.08 | 26.07 |
| **TB88** | 24.02 | 24.02 | 24.95 | 20.75 | 20.77 | 20.72 | 24.80 | 24.81 | 24.80 |
| **TB89** | 24.89 | 24.90 | 24.49 | 22.02 | 22.04 | 22.00 | 26.06 | 26.08 | 26.06 |
| **TB90** | 24.15 | 24.19 | 24.47 | 22.17 | 22.19 | 22.14 | 26.14 | 26.15 | 26.14 |
| **TB91** | 24.22 | 24.19 | 24.47 | 22.16 | 22.18 | 22.13 | 26.11 | 26.12 | 26.11 |
| **TB92** | 24.76 | 24.80 | 24.47 | 22.23 | 22.25 | 22.21 | 26.26 | 26.27 | 26.26 |
| **TB93** | 24.16 | 24.17 | 24.46 | 22.21 | 22.23 | 22.19 | 26.27 | 26.28 | 26.27 |
| **TB94** | 24.24 | 24.21 | 24.46 | 22.23 | 22.25 | 22.21 | 26.34 | 26.35 | 26.34 |
| **TB95** | 24.97 | 24.95 | 24.46 | 22.24 | 22.27 | 22.22 | 26.38 | 26.39 | 26.38 |
| **TB96** | 24.42 | 24.45 | 24.45 | 22.30 | 22.32 | 22.27 | 26.37 | 26.39 | 26.37 |
| **TB97** | 24.96 | 24.95 | 24.43 | 22.36 | 22.38 | 22.33 | 26.39 | 26.40 | 26.39 |
| **TB98** | 24.74 | 24.76 | 24.42 | 22.44 | 22.47 | 22.42 | 26.41 | 26.42 | 26.41 |
| **TB99** | 24.16 | 24.17 | 24.95 | 20.74 | 20.76 | 20.72 | 24.80 | 24.81 | 24.80 |
| **TB100** | 24.79 | 24.78 | 24.42 | 22.37 | 22.39 | 22.35 | 26.52 | 26.53 | 26.52 |
| **TB101** | 24.13 | 24.12 | 24.41 | 22.42 | 22.44 | 22.39 | 26.52 | 26.53 | 26.52 |
| **TB102** | 24.35 | 24.38 | 24.41 | 22.46 | 22.48 | 22.43 | 26.47 | 26.48 | 26.47 |
| **TB103** | 24.18 | 24.21 | 24.40 | 22.48 | 22.51 | 22.46 | 26.62 | 26.63 | 26.62 |
| **TB104** | 24.83 | 24.80 | 24.40 | 22.50 | 22.52 | 22.47 | 26.62 | 26.63 | 26.62 |
| **TB105** | 24.94 | 24.95 | 24.40 | 22.55 | 22.57 | 22.53 | 26.62 | 26.63 | 26.62 |
| **TB106** | 24.99 | 24.98 | 24.40 | 22.52 | 22.55 | 22.50 | 26.63 | 26.64 | 26.63 |
| **TB107** | 24.41 | 24.38 | 24.39 | 22.62 | 22.64 | 22.60 | 26.73 | 26.74 | 26.73 |
| **TB108** | 24.48 | 24.50 | 24.39 | 22.68 | 22.70 | 22.66 | 26.86 | 26.88 | 26.86 |
| **TB109** | 24.54 | 24.55 | 24.39 | 22.76 | 22.78 | 22.74 | 26.86 | 26.88 | 26.86 |
| **TB110** | 24.84 | 24.80 | 24.95 | 20.76 | 20.79 | 20.74 | 24.85 | 24.86 | 24.85 |
| **TB111** | 24.11 | 24.12 | 24.38 | 22.68 | 22.70 | 22.66 | 26.78 | 26.79 | 26.78 |
| **TB112** | 24.65 | 24.62 | 24.38 | 22.74 | 22.77 | 22.72 | 26.80 | 26.81 | 26.80 |
| **TB113** | 24.51 | 24.50 | 24.37 | 22.65 | 22.67 | 22.63 | 27.02 | 27.03 | 27.02 |
| **TB114** | 24.99 | 24.96 | 24.37 | 22.76 | 22.78 | 22.74 | 27.04 | 27.05 | 27.04 |
| **TB115** | 24.92 | 24.95 | 24.37 | 22.91 | 22.94 | 22.89 | 27.21 | 27.23 | 27.21 |
| **TB116** | 24.57 | 24.58 | 24.36 | 22.91 | 22.94 | 22.89 | 27.21 | 27.23 | 27.21 |
| **TB117** | 24.58 | 24.58 | 24.36 | 22.93 | 22.96 | 22.91 | 27.17 | 27.18 | 27.17 |
| **TB118** | 24.59 | 24.62 | 24.35 | 22.95 | 22.98 | 22.93 | 27.11 | 27.12 | 27.11 |
| **TB119** | 24.98 | 24.97 | 24.35 | 22.86 | 22.88 | 22.84 | 27.09 | 27.10 | 27.09 |
| **TB120** | 24.60 | 24.63 | 24.33 | 22.86 | 22.88 | 22.84 | 27.46 | 27.47 | 27.46 |
| **TB121** | 24.06 | 24.06 | 24.93 | 20.77 | 20.79 | 20.75 | 24.85 | 24.86 | 24.85 |
| **TB122** | 24.89 | 24.87 | 24.32 | 22.90 | 22.92 | 22.87 | 27.43 | 27.44 | 27.43 |
| **TB123** | 24.55 | 24.55 | 24.31 | 22.88 | 22.90 | 22.86 | 27.41 | 27.42 | 27.41 |
| **TB124** | 24.93 | 24.96 | 24.31 | 23.11 | 23.14 | 23.09 | 27.81 | 27.82 | 27.81 |
| **TB125** | 24.97 | 24.98 | 24.31 | 23.16 | 23.18 | 23.13 | 28.50 | 28.51 | 28.50 |
| **TB126** | 24.95 | 24.97 | 24.30 | 23.37 | 23.39 | 23.35 | 28.85 | 28.86 | 28.85 |
| **TB127** | 24.31 | 24.34 | 24.29 | 23.40 | 23.42 | 23.37 | 28.85 | 28.86 | 28.85 |
| **TB128** | 24.12 | 24.13 | 24.28 | 23.42 | 23.45 | 23.40 | 28.71 | 28.73 | 28.71 |
| **TB129** | 24.79 | 24.78 | 24.27 | 23.45 | 23.47 | 23.43 | 28.71 | 28.73 | 28.71 |
| **TB130** | 24.66 | 24.63 | 24.26 | 23.45 | 23.47 | 23.43 | 28.71 | 28.73 | 28.71 |
| **TB131** | 24.53 | 24.54 | 24.25 | 23.69 | 23.71 | 23.67 | 28.71 | 28.73 | 28.71 |

|  | **negative AFB smear staining/sputum culture** | | | | | | | | |  | **positive AFB smear staining/sputum culture** | | | | | | | | |
| --- | --- | --- | --- | --- | --- | --- | --- | --- | --- | --- | --- | --- | --- | --- | --- | --- | --- | --- | --- |
|  | **cel-miR-39** | | | **ASdes** | | | **MTBmiR-5** | | |  | **cel-miR-39** | | | **ASdes** | | | **MTBmiR-5** | | |
|  | **#1** | **#2** | **#3** | **#1** | **#2** | **#3** | **#1** | **#2** | **#3** |  | **#1** | **#2** | **#3** | **#1** | **#2** | **#3** | **#1** | **#2** | **#3** |
| **Patient 1** | 24.14 | 24.59 | 24.34 | 24.86 | 24.85 | 24.98 | 28.42 | 28.36 | 28.93 |  | 24.90 | 24.50 | 24.55 | 25.31 | 25.31 | 25.51 | 26.92 | 27.47 | 27.30 |
| **Patient 2** | 24.74 | 24.83 | 24.27 | 25.01 | 25.83 | 25.09 | 29.05 | 29.26 | 29.64 |  | 24.75 | 24.32 | 24.62 | 25.48 | 25.46 | 25.34 | 24.74 | 24.52 | 24.41 |
| **Patient 3** | 24.71 | 24.02 | 24.69 | 27.15 | 27.20 | 27.04 | 29.10 | 29.11 | 29.89 |  | 24.53 | 24.28 | 24.12 | 20.82 | 20.71 | 20.78 | 24.52 | 24.81 | 24.98 |
| **Patient 4** | 24.89 | 24.30 | 24.27 | 23.20 | 23.88 | 23.54 | 27.72 | 27.69 | 27.64 |  | 24.86 | 24.12 | 24.52 | 21.53 | 21.00 | 20.73 | 24.66 | 24.51 | 24.51 |
| **Patient 5** | 24.61 | 24.45 | 24.56 | 21.77 | 21.72 | 21.03 | 25.48 | 25.88 | 25.66 |  | 24.31 | 24.54 | 24.70 | 20.05 | 20.77 | 20.26 | 24.47 | 24.97 | 24.74 |
| **Patient 6** | 24.00 | 24.10 | 24.44 | 23.06 | 23.70 | 23.53 | 27.39 | 27.34 | 27.01 |  | 24.97 | 24.08 | 24.43 | 23.12 | 23.91 | 23.44 | 27.09 | 27.34 | 27.63 |
| **Patient 7** | 24.85 | 24.19 | 24.13 | 27.11 | 27.29 | 27.17 | 29.69 | 29.84 | 29.55 |  | 24.73 | 24.10 | 24.75 | 20.59 | 20.52 | 20.41 | 24.60 | 24.61 | 24.79 |
| **Patient 8** | 24.59 | 24.78 | 24.45 | 23.59 | 23.57 | 23.06 | 27.52 | 27.48 | 27.22 |  | 24.57 | 24.43 | 24.08 | 21.66 | 21.63 | 21.98 | 24.47 | 24.80 | 24.91 |
| **Patient 9** | 24.24 | 24.50 | 24.84 | 25.71 | 25.65 | 25.46 | 30.75 | 30.55 | 30.90 |  | 24.67 | 24.99 | 24.25 | 20.24 | 20.53 | 20.84 | 29.44 | 29.51 | 29.68 |
| **Patient 10** | 24.97 | 24.34 | 24.53 | 29.71 | 29.90 | 28.83 | 30.12 | 30.97 | 30.87 |  | 24.31 | 24.59 | 24.57 | 23.54 | 23.67 | 23.70 | 28.24 | 28.77 | 29.00 |
